# Molecular Epidemiology of *Streptococcus pneumoniae* Isolates from Children with Recurrent Upper Respiratory Tract Infections

**DOI:** 10.1371/journal.pone.0158909

**Published:** 2016-07-14

**Authors:** Izabela Korona-Glowniak, Maciej Maj, Radosław Siwiec, Artur Niedzielski, Anna Malm

**Affiliations:** 1 Department of Pharmaceutical Microbiology with Laboratory for Microbiological Diagnostics, Medical University of Lublin, Lublin, Poland; 2 Department of Clinical Immunology, Medical University of Lublin, Lublin, Poland; 3 Otoneurology Lab III Chair of Paediatric, Medical University of Lublin, Lublin, Poland; 4 Department of Pediatric Otolaryngology, Phoniatrics and Audiology, Medical University of Lublin, Lublin, Poland; Instituto Butantan, BRAZIL

## Abstract

A total of 125 isolates were recovered from adenoids and/or nasopharynx of 170 children aged 2 to 5 from south-east Poland; they had undergone adenoidectomy for recurrent and/or persistent symptoms of upper respiratory tract infections. Pneumococcal isolates were analyzed by phenotyping (serotyping and antimicrobial resistance tests) and genotyping together with the clonality of the pneumococcal isolates based on resistance determinants, transposon distribution and multilocus sequence typing (MLST). Serotypes 19F, 6B and 23F constituted 44.8% of the isolates. Among all of the strains, 44.8% showed decreased susceptibility to penicillin and resistance to co-trimoxazole (52.8%), tetracycline (38.4%), erythromycin (53.6%), clindamycin (52.8%) and chloramphenicol (27.2%) was observed. Tn*6002* was found in 34.8% of erythromycin-resistant isolates while composite Tn*2010*—in 16.7% of *erm*(B)-carrying isolates that harboured also *mef*(E) gene. Tn*3872*-related elements were detected in 27.3% of erythromycin-resistant strains. In the majority of chloramphenicol-resistant *cat*_pC194_-carrying isolates (79.4%), ICESp23FST81-family elements were detected. The genotyping showed that pneumococcal population was very heterogeneous; 82 sequence types (STs) were identified, and the most frequent contributed to not more than 8% of the isolates. Nearly 44% STs were novel, each of them was recovered only from one child. Four STs belonged to one of the 43 worldwide spread resistant pneumococcal clones currently accepted by Pneumococcal Molecular Epidemiology Network (PMEN), i.e. Spain 9V-3, Spain 23F-1, Norway NT-42 and Poland 6B-20, accounting for 12 (16.7%) of the 75 nonususceptible isolates, and five STs were single-locus variants of PMEN resistant clones (England 14–9, Spain 9V-3, Spain 23F-1, Greece 21–30, Denmark 14–32), accounting 9 (12%) of nonsusceptible isolates. A few MDR clones belonging to 6B and 19F serotypes found among preschool children emphasizes rather the role of clonal dissemination of local strains in the community than international clones spreading in the increase of resistance among pneumococcal strains.

## Introduction

*Streptococcus pneumoniae* is an important etiologic agent of meningitis, pneumonia, bacteriemia and acute otitis media in both children and adults and one of the major bacterial pathogens colonizing nasopharynx, mainly asymptomatically [1]. Young children are colonized most frequently and they have been found to be the main reservoir of pneumococci, paying the key role in spreading and selecting multidrug resistant strains [2]. It was shown that adenoids play a crucial role in aetiology of otitis media, rhinosinusitis and adenotonsilitis and *S*. *pneumoniae* is one of the most frequent pathogen detected in adenoids where it gives rise to chronic infection, swelling and inflammation [3].

Recently, there has been an increasing rate of antibiotic resistance in the pneumococcal serotypes that are responsible for the infections of a middle ear, nasal cavity and pharynx in children and cause difficulties in the treatment [4]. Multiple resistance of pneumococci especially resistance to macrolides (*erm*(B) and/or *mef*(A/E)) and tetracyclines (*tet*(M)) as well as to chloramphenicol (*cat*) is generally associated with their unique recombination-mediated genetic plasticity and possessing the mobile genetic elements, including those of Tn*916* and Tn*5252* families [5]. Pneumococcal resistance to erythromycin and tetracycline is associated with the insertion of the *erm*(B) into the transposons of the Tn*916* or Tn*917* family, include Tn*6002*, Tn*1545* (carrying also the kanamycin resistance gene *aph3-III*), and Tn*3872* (carrying also transposase genes *tnpA* and *tnpR*) [5]. Recently two new composite elements of the Tn*916* family, containing *tet*(M) plus MEGA (macrolide efflux genetic assembly) carrying *mef*(E) gene (Tn*2009*) and *tet*(M), *erm*(B) and MEGA (Tn*2010*) have been described [6, 7]. Resistance to chloramphenicol in *S*. *pneumoniae* encoded by *cat* gene, which is carried on the Tn*5253*-like, a composite structure made up of two independent conjugative transposons. Besides, Tn*916*-like *tet*(M)-carrying element designated Tn*5251*, was inserted within the Tn*5252* element that carries chloramphenicol resistance [8].

Pneumococcal infections contribute to a large number of medical care visits and antibiotic prescriptions despite the advances in the development of pneumococcal conjugate vaccines (PCVs) leading to a reduction of invasive disorders both in infants, older children and adults. An additional benefit of the vaccine was a decrease in rates of antimicrobial resistance among pneumococcal isolates resulting from association resistance to penicillin, macrolides and multidrug resistance with serotypes 6B, 9V, 14, 19F and 23F included to PCVs [9]. Only in countries with routine effective use of PCV7, prevalence of infections caused by serotypes belonging to PCV7 decreased in comparison to the pre-vaccine era [10].

Many studies have revealed that worldwide pneumococcal diseases are mostly caused by a few multidrug-resistant clones [11, 12, 13, 14]. The aim of this study was to seek for the clonality among the pneumococcal strains detected in preschool children with recurrent upper respiratory tract infections (URTIs), based on the analysis of serotypes, antimicrobial susceptibility patterns and genotypic characteristics, including the transposons with resistance genes and clonality of the pneumococcal isolates performed by multilocus sequence typing (MLST) method.

## Materials and Methods

### Study population

The study enrolled 170 children, aged between 2 and 5, undergoing adenoidectomy in Department of Pediatric Otolaryngology, Phoniatrics and Audiology, Medical University of Lublin during May-June and November-December 2011 as well as May-June and October-December 2012. The indication for adenoidectomy was recurrent acute pharyngotonsilitis for at least 2 years with 5 or more acute attacks per year. Patients didn’t received any antibiotic therapy for at least 20 days before the operation. From all children’s parents, the written informed consent were obtained. None of the children were immunized by a pneumococcal vaccine. The Ethical Committee of the Medical University of Lublin approved the study protocol (No. KE-0254/75/211).

### Laboratory procedures

Before adenoidectomy, the nasopharyngeal specimens were obtained with sterile alginate-tipped swabs on aluminium shafts. After the surgery, the adenoid were placed in the sterile container and were transported to laboratory then the adenoid was swabbed with sterile alginate-tipped applicator. Swabs were inoculated on selective Mueller-Hinton agar with 5% sheep blood and 5 mg/L of gentamicin for selective cultivation of streptococci. The streaked agar plates were incubated aerobically at 35°C in 5% CO_2_ enriched atmosphere for 24 to 48 hours. Pneumococci were identified by colony morphology, susceptibility to optochin (5 μg), and bile solubility; identification was confirmed by a slide agglutination test Slidex Pneumo-Kit (BioMerieux).

All isolates were serotyped by means of Quellung reaction using antisera provided by Statens Serum Institute (Copenhagen, Denmark). The isolates nontypeable (NT) were confirmed by the restriction digest (BsaI) of PCR product of *lytA* gene encoding the autolysin enzyme specific to *S*. *pneumoniae* [15].

Susceptibility of the isolates to oxacillin, erythromycin (E), tetracycline (Te), chloramphenicol (C), clindamycin (Cc), Norfloxacin (Nor), rifampicin (Ra), teicoplanin (Tec), linezolid (Lzd) and trimethoprim-sulfamethoxazole (Sxt) was determined by the disk diffusion method of Bauer and Kirby on Mueller-Hinton agar with 5% mechanically defibrinated horse blood and 20 mg/L β-NAD. Results were interpreted according to the European Committee on Antimicrobial Susceptibility Testing recommendations (EUCAST, 2011). Isolates exhibiting a zone of ≥ 20 mm around a 1 μg oxacillin disk were reported as penicillin susceptible *S*. *pneumoniae* (PSSP); isolates exhibiting a zone of < 20 mm were further tested by the E-test (AB Biodisk, Sweden), following the manufacturer’s instruction, to determine minimal inhibitory concentration (MIC) for benzylpenicillin. Isolates with MIC ≤ 0.064 mg/L were considered as fully susceptible to benzylpenicillin; isolates with MIC > 0.064 mg/L were called penicillin non-susceptible *S*. *pneumoniae* (PNSSP). Multidrug-resistant isolates of *S*. *pneumoniae* (MDR-SP) were defined as having resistance to at least 3 different classes of antibiotics. *S*. *pneumoniae* ATCC 49619 was used as control strain in the antimicrobial susceptibility tests.

### Amplification experiments and gene detection

Bacterial genomic DNA were prepared with Genomic Mini Kit (A&A Biotechnology, Gdynia, Poland) and were used as templates for PCR. Macrolide resistance genes *erm*(B) and *mefA/E* were detected by PCR using the primers and conditions described previously [16]. The PCR product of the *mefA/E* gene were digested with *BamH*I (Fermentas) to differentiate between the *mef*(A) and *mef*(E) gene subclasses [17]. PCR amplification was used to detect cat_pC194_ gene related to chloramphenicol resistance [18]. The Tn*916* and Tn*917* transposon-related genes (*int916*, *xis916*, *tnpA* and *tnpR*, O13-O14), the tetracycline resistance gene, *tet*(M), and the promoter of the kanamycin resistance gene *aph3-III* were detected by PCR using the primers and conditions described previously [19, 20]. PCR with primer pair J12/J11was used to distinguish by size of region *orf20* to *orf19*, among Tn*3872*, Tn*6002* and Tn*6003*/Tn*1545*, which yield amplicons of 0.8 kb, 3.7 kb, and 7.9 kb, respectively [19]. An *erm*(B)/*tet*(M) linkage was detected using the primers described previously [19]. The O_6_/TET2dg amplicon of putative IS1239 insertion was used to distinguished Tn*6003* and Tn*1545* [19]. The *mef*(E)-positive isolates were analyzed for the presence Tn*2010*-like element by PCR with primers *tetM*1-OM21, *msrA*2-SG3, OM18-*xis*R detecting MEGA element [21]. REDTaq ReadyMix (Sigma-Aldrich) was used in standard PCR and Long PCR Enzyme Mix (Thermo-Scientific) was used in reaction expected to yield PCR products exceeding 3 kb in size. Tn*5252* was detected by PCR of its transposase gene, *int5252* and excisionase *xis5252* [8]. For the detection of Tn*5253*, the region of the right junction between Tn*5251* and Tn*5252* was analyzed [22]. Primers for the detection of ICESp23FST81 have been described elsewhere [8]. PCR products of resistance genes and transposon markers related to presumptive transposons were presented in Table 1.

**Table 1:** PCR products of resistance genes and transposon markers related to presumptive transposons.

	Resistance genes					Tn*916*-family transposons						Tn*5252*-family transposons		
Presumptive transposon	*tet*(M)	*erm*(B)	*mef*(E)	*aph3*-III	*cat*_pC194_	*int/xis*916	*tnp*A/*tnp*R	*Orf9*^a^	*Orf20-19*^b^ amplicons	IS*1239*^c^	MEGA element^d^	*int*/*xis* Tn5252	*Jun* Tn5253	*intICE*
Tn*916*	+	-	-	-		+/+	-/-	+	0.8 kb	-	-			
Tn*6002*	+	+	-	-		+/+	-/-	+	3.7 kb	-	-			
Tn*6003*	+	+	-	+		+/+	-/-	+	7.9 kb	-	-			
Tn*1545*	+	+	-	+		+/+	-/-	+	7.9 kb	+	-			
Tn*3872*	+	+	-	-		+/+	+/+	-	0.8 kb	-	-			
Tn*2009*	+	-	+	-		+/+	-/-	-	0.8 kb	-	+			
Tn*2010*	+	+	+	-		+/+	-/-	-	0.8 kb	-	+			
Tn*2017*	+	+	+	-		+/+	+/+	-	0.8 kb	-	+			
Tn*5252*					+							+/+	-	-
Tn*5253*-like					+							+/+	+	-
ICE*Sp23FST81*					+							-/-	-	+

^a^ using O13-O14 primers [20]

^b^ using J12/J11 primers [19]

^c^ using O_6_/TET2dg primers [19]

^d^ using *tetM*1-OM21/*msrA*2-SG3/OM18-*xis*R primers [21]

### Multilocus sequence typing (MLST) analysis

MLST was performed as described previously [23]. The internal fragments of 7 housekeeping genes (*aroE*, *gdh*, *gki*, *recP*, *spi*, *xpt*, *ddl*) were amplified from chromosomal DNA by PCR methods for all of 125 isolates. DNA sequences were determined by the dideoxy-chain termination method using an automatic DNA analyser (LICOR 4300), the USB Thermo Sequenase Cycle Sequencing Kit (Affymetrix), and IRD 800- and IRD700-labeled custom sequencing primers. Sequences were determined on both strands using denatured double-stranded DNA templates. The sequences types (STs) were determined by the comparison with those of corresponding allelic profiles at MLST database (http://spneumoniae.mlst.net). The new STs and alleles were submitted to the curator of MLST website for assignments. eBURSTv3 software available at the MLST website (http://www.eburst.mlst.net) was used to explore the similarity among tested isolates as well as correlation between the STs of tested isolates with all STs existent in MLST database. Strains were grouped in the same clonal group (CG) when six or more of the seven loci were identical and clonal complexes (CC) were assigned by comparing tested strain collection MLST data with whole MLST database.

## Results

A total of 125 isolates were recovered from adenoids and/or nasopharynx of 170 children aged 2 to 5 who had undergone adenoidectomy for recurrent and/or persistent symptoms of URTIs.; 16 (9.4%) children were colonized by more than two different in colony morphology isolates (14 and 2 children were colonized by 2 and 3 pneumococcal strains, respectively), which were identified by phenotyping and genotyping as different pneumococcal strains. Sixty six (52.8%) *S*. *pneumoniae* strains colonized both the nasopharynx and the adenoid core of children, 42 (33.6%) strains were obtained only from the adenoid core and 17 (13.6%) strains only from the nasopharynx one. Pneumococcal colonization was observed in 107 (62.9%) children.

### Serotype distribution and vaccine coverage

The results of serotyping and antimicrobial resistance for 62 pneumococcal strains were already presented elsewhere [24]. Twenty three different serotypes were found and 6 isolates (4.8%) were nontypeable (NT). Serotypes 19F, 6B and 23F constituted 44.8% of the isolates. Serotypes belonging to pneumococcal conjugated vaccines—PCV10 and PCV13 constituted 54.4% and 66.4% of the isolates, respectively.

### Antimicrobial susceptibility

**Fig 1:**
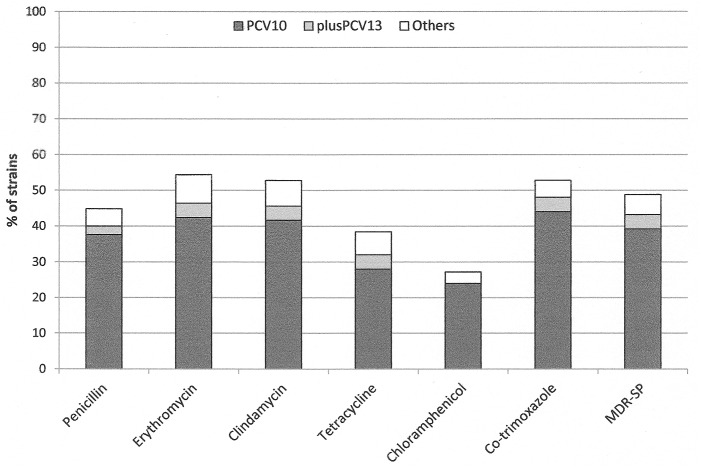
Resistance rate to antibiotics of *Streptococcus pneumoniae* isolated from children undergoing adenoidectomy for recurrent and/or persistent symptoms of URTIs. Dark blocks, samples containing PCV10 serotypes (1, 4, 5, 6B, 7F, 9V, 14, 18C, 19F, 23F); grey blocks, samples containing serotypes in PCV13 (3, 6A, 19A additionally to 10-valent vaccine); white blocks, samples containing other serotypes.

The pneumococcal isolates were susceptible to all tested antimicrobial agents in 40.0%. These strains belonged to serogroup 15 (10 isolates), serotypes 3 (9 isolates), 11A (4 isolates), 35F (4 isolates), 38 (3 isolates), 6A, 6B, 14, 19F, 23A, 23B (2 isolates per each serotype), and 10A, 16F, 18C, 22F, 23F, 31, 34, 35C (1 isolate per each serotype). Among all of the strains, 44.8% showed decreased susceptibility to penicillin (MIC range 0.12–2.0 mg/L, MIC_50_ 0.5 mg/L and MIC_90_ 2.0 mg/L). *S*. *pneumoniae* isolates were resistant to co-trimoxazole (52.8%), tetracycline (38.4%), erythromycin (53.6%), clindamycin (52.8%) and chloramphenicol (27.2%) (Fig 1). All isolates were susceptible to levofloxacin and moxifloxacin. None of the tested isolates was resistant to rifampicin, linezolid and teicoplanin. Multidrug resistance was present in 48.8% of the isolates. Among MDR-SP 85.3% were non-susceptible to penicillin. Antibiotic resistant pneumococci were mostly distributed among serotypes belonging to PCV10 and PCV13 (Fig 1). PNSSP and MDR-SP strains represented PCV10 serotypes in 83.9% and 80.3%, respectively and PCV13 serotypes in 89.3% and 88.5%, respectively. Colonization with PNSSP and MDR-SP strains was found in 54 (31.8%) and 58 (34.1%) children, respectively.

### Detection of resistance genes

Resistance determinants were detected in all of 71 strains with phenotype of resistance to one or more of tetracycline (Te), chloramphenicol (C), erythromycin (E) and clindamycin (Cc). All of the tetracycline-resistant strains possessed the *tet*(M) gene. The presence of the *tet*(M) gene was found in 20 tetracycline-sensitive strains as well. All isolates with erythromycin and clindamycin resistance phenotype had the *erm*(B) gene. The *mef*(E) gene, but not the *mef*(A), was also found in 11 isolates with the *erm*(B) gene. The *mef*(E) gene was only detected in the erythromycin resistant but not in the clindamycin-sensitive strain. Twelve strains positive for the promoter of the *aph3’* gene were found. Each of the 34 strains with resistance to chloramphenicol was positive for the cat_pC194_ gene.

### Transposon distribution

The strains with phenotype of resistance to one or more of tetracycline, chloramphenicol, erythromycin and clindamycin were graded according to resistance phenotype. There were following resistant patterns: E (1 strain), ECc (5 strains), ECcTe (31 strains), C (2 strains), TeC (2 strains), ECcC (15 strains) and ECcTeC (15 strains). Among pneumococcal strains with resistance pattern ECcTe, 10 contained *erm*(B), *tet*(M), *int/xis*916 genes related to Tn*6002*. Ten isolates were detected for *erm*(B), *tet*(M), *mef*(E), *int/xis*916 genes carrying Tn*2010*. Six isolates were positive for *erm*(B), *tet*(M), *int/xis*916, *tnp*A, *tnp*R genes related to Tn*3872*. Four isolates containing *erm*(B), *tet*(M), *int/xis*916 genes were also positive for the promoter of the aph3’ related to Tn*6003*. One isolate was positive for *erm*(B), *tet*(M) and *tndX*, indicating the presence of a related Tn*1116* transposon. Three strains with ECc pattern possessed Tn*6002* while Tn*6003* and Tn*3872* were found in two others (Table 2).

**Table 2:** Characteristics of 125 *S*. *pneumoniae* strains from children who had undergone adenoidectomy for recurrent and/or persistent symptoms of URTIs.

					Genes detected by PCR		Presumptive transposons		Related PMEN clone
CG^a^ (no of isolates)	Predicted founder ST/CC^b^	ST (no of isolates)	Serotype (no of isolates)	Resistance pattern (no of isolates)	Resistance genes	Transposon genes	Tn916 family elements	Tn5252-like elements	
**CG1 (8)**	15	423(4)	19F(4)	TeCSxt(1)	*tet(M)*, *cat*_*pC194*_	*int/xis916*, *intICE*	Tn*916*	ICE*Sp*23FST81-like	England 14–9 DLV
				ECcTe(1)	*tet(M)*, *erm(B)*	*int/xis916*	Tn*6002*		
				ECcTeCSxt(1)	*tet(M)*, *erm(B)*, *cat*_*pC194*_	*int/xis916*, *intICE*	Tn*6002*	ICE*Sp*23FST81-like	
				ECcTeCSxt(1)	*tet(M)*, *erm(B)*, *mef(E)*, *cat*_*pC194*_	*int/xis916*, *intICE*	Tn*2010*	ICE*Sp*23FST81-like	
		15(1)	14(1)	ECcTeSxt(1)	*tet(M)*, *erm(B)*	*int/xis916*, *tnpA/tnpR*	Tn*3872*		England 14–9 SLV
		1815(1)	19F(1)	CSxt(1)	*cat*_*p194*_			None (pC194)	
		9251(1)	19F(1)	ECcTeSxt(1)	*tet(M)*, *erm(B)*	*int/xis916*	Tn*6002*		
		721(1)	19F(1)	ECcTeSxt(1)	*tet(M)*, *erm(B)*, *mef(E)*	*int/xis916*	Tn*2010*		England 14–9 DLV
**CG2 (6)**	87	87(3)	19F(6)	PECcTeCSxt(6)	*tet(M)*, *erm(B)*, *cat*_*pC194*_	*int/xis916*, *int/xis5252*	Tn*6002*	Tn*5252*	
		10327(1)			*tet(M)*, *erm(B)*, *cat*_*pC194*_	*int/xis916*, *int/xis5252*	Tn*6002*	Tn*5252*	
		9253(1)			*tet(M)*, *erm(B)*, *cat*_*pC194*_	*int/xis916*, *int/xis5252*	Tn*6002*	Tn*5252*	
		9268(1)			*tet(M)*, *erm(B)*, *cat*_*pC194*_	*int/xis916*, *int/xis5252*	Tn*6002*	Tn*5252*	
**CG3 (6)**	156	156(2)	14(1)	PECcTeSxt(1)	*tet(M)*, *erm(B)*	*int/xis916*	Tn*6002*		Spain 9V-3
			9V(1)	PSxt(1)					
		3811 (2)	15A (2)	S (2)					Spain 9V-3 SVL
		9258 (1)	15A (1)	S (1)					Spain 9V-3 DVL
		9269 (1)	9V(1)	PSxt (1)					Spain 9V-3 SVL
**CG4 (3)**	433	433(1)	22F (1)	S					
		9259(1)	22F(1)	ECcTe (1)	*tet(M)*, *erm(B)*	*int/xis916*	Tn*6002*		
		9272(1)	35C (1)	S					
**CG5 (5)**	180	180 (3)	3 (5)	S (5)					Netherlands 3–31
		9254 (1)							Netherlands 3–31 SVL
		3794 (1)							Netherlands 3–31 SVL
**CG6 (3)**	8991	8991(1)	NT(3)	PECcTeSxt(1)	*tet(M)*, *erm(B)*, *aph3-III*	*int/xis916*	Tn*6003*		Norway NT-42 DVL
		9273(1)		PECcTeSxt(1)	*tet(M)*, *erm(B)*, *aph3-III*	*int/xis916*	Tn*6003*		
		9270(1)		PECcTeCSxt(1)	*tet(M)*, *erm(B)*, *aph3-III*, *cat*_*pC194*_	*int/xis916*	Tn*6003*	ICE*Sp*23FST81-like	
**CG7(9)**	81	81(7)	23F (9)	PECcCSxt(2)	*tet(M)*, *erm(B)*, *cat*_*p194*_	*int/xis916*, *intICE*	Tn*6002*	ICE*Sp*23FST81-like	Spain 23F-1
				PECcCSxt(5)	*tet(M)*, *erm(B)*, *aph3-III*, *cat*_*pC194*_	*int/xis916*, *intICE*	Tn*6003*	ICE*Sp*23FST81-like	
		2033(1)		PECcTeCSxt(1)	*tet(M)*, *erm(B)*, *aph3-III*, *cat*_*pC194*_	*int/xis916*, *intICE*	Tn*6003*	ICE*Sp*23FST81-like	Spain 23F-1 SVL
		932(1)		PECcTeCSxt(1)	*tet(M)*, *erm(B)*, *aph3-III*, *cat*_*pC194*_	*int/xis916*, *intICE*	Tn*1545*	ICE*Sp*23FST81-like	Spain 23F-1 SVL
**CG8(3)**	439	42 (1)	23B (1)	S					Tennessee 23F-4 DLV
		439 (1)	23A (1)	S					Tennessee 23F-4 SLV
		9264(1)	23A(1)	ECcTeC(1)	*tet(M)*, *erm(B)*, *cat*_*p194*_	*int/xis916*, *intICE*	Tn*6002*	ICE*Sp*23FST81-like	Tennessee 23F-4 DLV
**CG9(3)**	156	4576(1)	14 (1)	PE(1)	*mef(E)*		MEGA		
		143(1)	14(1)	PECcSxt (1)	*erm(B)*	*int/xis916*, *tnpA/tnpR*	Tn*3872*		Spain 9V-3 DVL
		10336(1)	19F (1)	PSxt (1)					
**CG10 (3)**	460	446 (1)	35F (3)	S (3)					
		9271 (1)							
		4052 (1)							
**CG11(8)**	320	320(7)	19F(7)	PECcTeSxt(7)	*tet(M)*, *erm(B)*, *mef(E)*	*int/xis916*	Tn*2010*		Taiwan19F-14 DVL
		2477(1)	19F(1)	PECcTeSxt(1)	*tet(M)*, *erm(B)*, *mef(E)*	*int/xis916*	Tn*2010*		Taiwan19F-14 DVL
**CG12(11)**	473	135(10)	6B(10)	PECcCSxt(7)	*tet(M)*, *erm(B)*, *cat*_*pC194*_	*int/xis916*, *tnpA/tnpR*, *intICE*	Tn*3872*	ICE*Sp*23FST81-like	
				PECcTeCSxt(2)	*tet(M)*, *erm(B)*, *cat*_*pC194*_	*int/xis916*, *tnpA/tnpR*, *intICE*	Tn*3872*	ICE*Sp*23FST81-like	
				PECcTeSxt(1)	*tet(M)*, *erm(B)*	*int/xis916*, *tnpA/tnpR*	Tn*3872*		
		9255(1)	6B(1)	PECcCSxt(1)	*tet(M)*, *erm(B)*, *cat*_*pC194*_	*int/xis916*, *tnpA/tnpR*, *intICE*	Tn*3872*	ICE*Sp*23FST81-like	
**CG13 (3)**	180	2049(2)	3 (3)	S (3)					
		505(1)							Netherlands 3–31 DVL
**CG14 (2)**	156	124(1)	14 (2)	S (2)					Netherlands 14–35
		10335(1)							Netherlands 14–35 SVL
**CG15 (4)**	62	62(3)	11A (4)	S (4)					Netherlands 8-33DVL
		4478(1)							Netherlands 8-33DVL
**CG16 (3)**	393	393(2)	38 (3)	S (3)					
		10331(1)							
**Singletons**	None	10329 (1)	6B (1)	PECcTe(1)	*tet(M)*, *erm(B)*	*int/xis916*	Tn*6002*		
(45)	193	410(4)	15A (1)	S (1)					Greece 21–30 SLV
			15A(1)	TeC (1)	*tet(M)*, *cat*_*pC194*_	*int/xis916*, *intICE*	Tn*916*	ICE*Sp*23FST81-like	
			14(1)	PECcTeSxt(1)	*tet(M)*, *erm(B)*	*int/xis916*, *tnpA/tnpR*	Tn*3872*		
			19F(1)	PECcTeSxt(1)	*tet(M)*, *erm(B)*, *mef(E)*	*int/xis916*	Tn*2010*		
	230	319(2)	19A(2)	PECcTeSxt(2)	*tet(M)*, *erm(B)*	*int/xis916*	Tn*6002*		Denmark 14–32 SLV
	193	3684(2)	19F (1)	S (1)					Portugal 19F-21 DVL
			19F(1)	ECcTe(1)	*tet(M)*, *erm(B)*	*int/xis916*, *tnpA/tnpR*	Tn*3872*		
	344	344(2)	NT(2)	PECcTeSxt(2)	*tet(M)*, *erm(B)*, *aph3-III*	*int/xis916*	Tn*6003*		Norway NT-42
	717	4668(2)	33F(2)	ECc(2)	*tet(M)*, *erm(B)*	*int/xis916*	Tn*6002*		
	315	315 (1)	6B	ECcSxt	*tet(M)*, *erm(B)*	*int/xis916*	Tn*6002*		Poland 6B-20
	Singleton	10315 (1)	6A	ECcTeSxt	*tet(M)*, *erm(B)*	*int/xis916*	Tn*6002*		
	2315	2315 (1)	NT	PCSxt		*intICE*		ICE*Sp*23FST81-like	
	320	257 (1)	19F	ECcTeSxt	*tet(M)*, *erm(B)*	*int/xis916*, *tnpA/tnpR*	Tn*3872*		Taiwan 19F-14 DVL
	496	496 (1)	18C	ECc	*tet(M)*, *erm(B)*, *aph3-III*	*int/xis916*	Tn*6003*		
	Singleton	9267 (1)	6B	PECcTeCSxt	*tet(M)*, *erm(B)*	*int/xis916*, *tnpA/tnpR*, *intICE*	Tn*3872*	ICE*Sp*23FST81-like	
	242	9265 (1)	23F	PECcTeSxt	*tet(M)*, *erm(B)*	*int/xis916*, *tnpA/tnpR*	Tn*3872*		Taiwan 23F-15 DVL
	66	9263 (1)	6A	ECcTeSxt	*tet(M)*, *erm(B)*	*int/xis916*	Tn*6002*		
	63	1545 (1)	19F	PECcTeSxt	*tet(M)*, *erm(B)*	*tndX*	Tn*1116*		Sweden 15A-25 DVL
	Singleton	9260 (1)	6B	PECcTeSxt	*tet(M)*, *erm(B)*	*int/xis916*	Tn*6002*		
	199	199(2)	15B	S					Netherlands 15B-37
	66	9257	15	S					Tennessee 14–18 DVL
	Singleton	9256	23F	Sxt					
	Singleton	10321	16F	S					
	460	9252	10A	S					
	460	10318	35F	S					
	4878	10316	34	S					
	156	176	6B	S					
	1016	102	18C	S					
	439	36	23F	S					
	378	1377	3	S					
	None	1014	6A	S					
	72	72	19F	S					
	Singleton	10339	23B	S					
	439	10338	23A	S					
	Singleton	9266	15	S					
	156	10330	6A	S					
	Singleton	9262	6B	S					
	1025	9261	15	S					
	3548	1994	31	S					

New STs are underlined; P, penicillin; E, erythromycin; Cc, clindamycin; Te, tetracycline; C, chloramphenicol; Sxt, co-trimoxazol; S, sensitive to all tested antibiotics; NT, nontypeable strain.

^a^ CG, Clonal Groups were assigned after tested strain collection analysis;

^b^Predicted founders were assigned by comparing tested strain collection MLST data with whole MLST database.

*cat*_pC194_ gene of resistance to chloramphenicol was located on Tn*5252* transposon, detected by *int/xis*5252 genes, in 6 strains with ECcTeC pattern. Twenty seven remaining chloramphenicol-resistant strains possessed ICESp23FST81 element carrying *cat*_pC194_ gene and *int*_ICESp23FST81_ gene of integrase. Neither *int/xis*5252 gene nor *int*_ICESp23FST81_ gene was detected in one strain with C pattern possessing *cat*_pC194_ gene (Table 2).

Strains with ECcC resistance were positive for Tn*3872* (8 strains) Tn*6002* (2 strains) and Tn*6003* (5 strains) additionally to ICE*Sp*23FST81-like elements. Tn*6002*, Tn*3872*, Tn*6003/1545* and Tn*2010* were detected in ECcTeC pattern group in 8, 3, 3 and 1 strain, respectively.

### Multi locus sequence typing (MLST)

**Fig 2:**
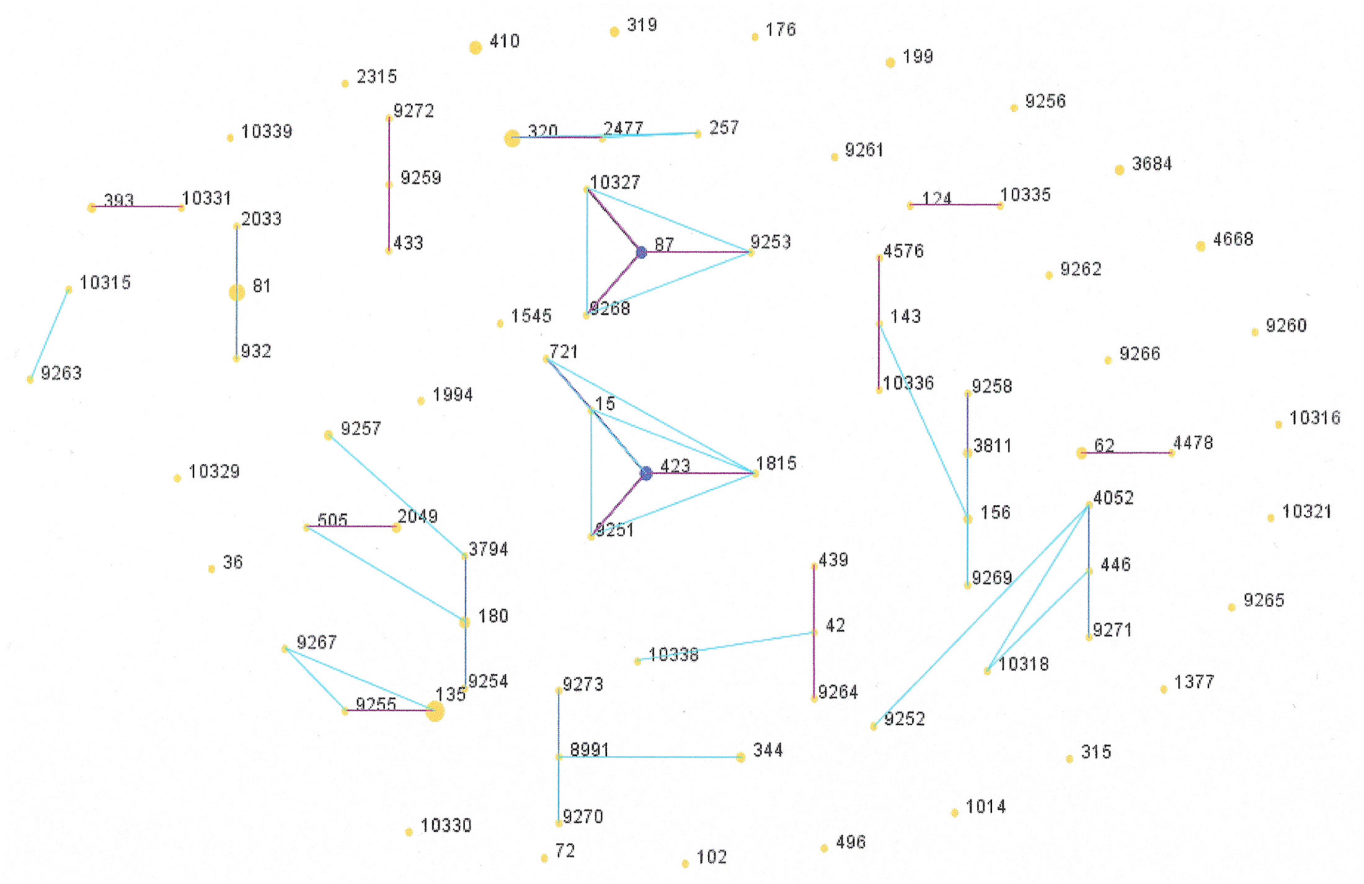
MLST-based population structure of Polish isolates obtained in 2011 to 2012, constructed using eBURST analysis (S1 File). The size of the circle (symbolizing STs) corresponds to the number of isolates belonging to a ST. Blue spots indicate the founder of a clonal group. Single-locus variant and double-locus variant links are represented by pink and blue lines, respectively.

Among 82 sequence types (STs) identified, 36 STs were newly assigned (ST8991, ST9251-9273, ST10315-10316, ST10318, ST10321, ST10327, ST10329-10331, ST10335-10336, ST10338-10339). Additionally, 3 new alleles (*gki398*, *gki457*, *spi393*) were assigned. Forty six STs with 80 isolates were grouped into 16 clonal groups, whereas 36 STs with 45 isolates were singletons (Fig 2, S1 File).

STs identical with seven of the 43 Pneumococcal Molecular Epidemiology Network (PMEN) clones were present in the Polish collection as well as fifteen single locus variants (SLVs) related to PMEN sequence (Table 2, S2 File, S1 Fig).

### Genotype in relation to serotype, antimicrobial resistance genes and transposons

For most of STs isolates represented by multiple isolates, only one serotype was identified. However, three STs were associated with two or three serotypes. These isolates were reserotyped in order to confirm the serotype. Each of the genotype-serotype associations in our study has previously been reported. A total of 43 STs were identified among nonsusceptible isolates, of which 18 were novel STs. Four STs belonged to one of the 43 worldwide spread resistant pneumococcal clones currently accepted by PMEN (i.e. Spain 9V-3, Spain 23F-1, Norway NT-42 and Poland 6B-20), accounting for 12 (16%) of the 75 nonususceptible isolates, and five STs were SLVs of PMEN resistant clones (England 14–9, Spain 9V-3, Spain 23F-1, Greece 21–30, Denmark 14–32), accounting 9 (12%) of nonsusceptible isolates (Table 2).

The penicillin-nonsusceptible isolates were heterogenous: 7 different capsule phenotypes (19F, 6B, 23F, 14, 9V, 19A and NT) were identified which belonged to 28 STs, including 14 novel STs, and were grouped in 7 clonal groups (CGs) and 9 singletons. Three STs belonged to PMEN clones (Table 2).

Similarity of the serotype, resistance genes and the transposon presence among strains belonging to the same CG were observed. Tn*5252* was detected in strains from CG2 only. Additionally, each of CG2 strains possessed Tn*6002*. All 8 strains from CG11 showed phenotype 19F PECcTeSxt and possessed Tn*2010* carrying the *mef*(E) gene. Transposon Tn*3872* was detected in 11 strains from CG12 and 10 strains possessed cat_pC194_ gene which was located on the ICE*Sp*23FST81-like element. GC7 consisted of 9 MDR strains showed 23F serotype and resistance genes and transposon markers typical for Tn*6003/1545* (7 strains) and Tn*6002* (2 strains). All of them possessed the ICESp23FST81-like element carrying cat_pC194_ gene. The most heterogenous group was CG1 (Table 2).

## Discussion

Our study is the first report describing the genotypic analysis of both antibiotic susceptible and resistant pneumococcal non-invasive isolates in Poland. These isolates were obtained from children who had undergone adenoidectomy for recurrent and/or persistent symptoms of URTIs were considered as non-invasive but causing chronically infected, swelling and inflamed adenoids. Our data suggest that PMEN clones are not widely distributed in non-invasive disease-associated isolates. Of the 16 clones, assigned to clonal groups, recovered in this study, all had previously been described in other countries, including 7 of the 43 worldwide-spread clones currently accepted by PMEN, which constituted 15.2% of isolated strains only. Additionally, 28 (34.1%) STs represented single-locus or double-locus variants (DLVs) of PMEN clones, and 7 of them were newly assigned. Such diverseness of the isolates was characteristic for pneumococcal strains collected from children attending day care centers in Norway [25]. Data presented by other authors were mostly related to invasive drug-resistant isolates and showed the vast predominance of PMEN clones [11, 26].

The possibility of comparison of the pneumococcal data obtained in different laboratories all around the world is the biggest achievement of global MLST database to monitor the molecular epidemiology of *S*. *pneumoniae* worldwide. In the present study, the prevalent STs were ST135, ST81 (Spain23F-1), ST320. These are multidrug resistant clones which are spread in many countries [12, 14, 27]. The most frequently CG isolated among antibiotic susceptible strains was CG5 (ST180/SLV180) currently accepted by PMEN as Netherlands 3–31; all isolates were of serotype 3. A representative isolate of Netherlands 14–35 and its SLV was also recognized as well as SLV and DLVs of Tennessee 23F-4 clone. Most isolates with *erm*(B)-mediated erythromycin resistance (71.6%) are also tetracycline-resistant, because of insertion of the *erm*(B) gene into conjugative transposons of the Tn*916*-family, which typically carry the *tet*(M) gene [5, 19, 20]. However, the presence of unexpressed *tet*(M) genes, found in tetracycline-sensitive strains, suggests that the Tn916-family transposons may be more widespread in *S*. *pneumoniae* than currently believed and should no longer be regarded as strictly associated with tetracycline resistance [20].

In our study, higher prevalence of Tn*3872* was related to the presence of clonal complex CG12 of ST135/SLV135 strains with 6B serotype. Interestingly, there was a connection between the serotype of isolates and the kind of transposable elements. Tn*6002* and Tn*2010* were detected mostly in 19F serotype, Tn3872 was detected in 6B serotype while among Tn*6003/1545* carriers most of them had 23F serotype or were nontypeable. Therefore, our results are indicative of the clonal dissemination process of transposable elements rather than the horizontal transfer which importance could be minor. However, detection in the strains related to PMEN clones more than one transposon that could be a result of horizontal gene transfer was described by others [14, 27].

The presence of Tn*2010* in 72.2% of macrolide resistant isolates from China was detected and CC271 strains carrying the Tn*2010* element expressing the high-level resistance to erythromycin were predominant in China [21]. In our study, the majority of strains possessing Tn*2010* (73%) belonging to CG11 clone consisted of ST320 and its SLV variant ST2477. ST271 and ST320 belonged to serotypes 19A or 19F, within the same clonal complex [12, 14] and in both of them Tn*2010* was detected. This may indicate that some genotypes are especially predisposed to be recipients of resistance genes or composite elements such as Tn*2010*, which has been found in members of this genetic lineage [7]. Interestingly, after the introduction of the PCV7, the genotype ST320, related to a multidrug-resistant internationally prevalent clone, Taiwan19F-14 (that also carries *mef*A/*erm*B determinants) was identified in invasive MDR 19A isolates in the United States and Spain [28,29]. In Canada, ST320, was associated with serotype 19F prior to the PCV7 introduction, whereas post-PCV7, ST320 has emerged as dominant among 19A isolates [30]. The fact that, in our study, all of ST320 isolates belonged to 19F serotype could result from a low level of vaccination in Poland. However, serotype 19A was increasingly recognized in Korean children before the introduction of PCV7 and ST320 was the most common ST (90%) among serotype 19A and in only 9% of serotype 19F isolates [31]. Choi et al. [31] suggested that ST320 has a selective advantage in serotype 19A strains and that further studies are necessary to explain why certain STs exhibit different selective pressures according to the serotype.

A routine antipneumococcal vaccination had not been implemented in Poland yet. Currently, in Poland both 23-valent polysaccharide vaccine for children ≥ 2 years old and adults as well as PCV10 and PCV13 for children ≤ 2 years old have been recommended in the national immunization schedule. The vaccination is administered on parental request and is paid for by parents. Since 2010, PCVs have been approved for use with children exhibiting a higher risk of pneumococcal disease and is covered by health insurance. According to National Institute of Public Health of National Institute of Hygiene annual data, between 2007 and 2014 years the number of vaccinated children aged 0–14 years old increased from 1.5% to 4% [32]. Our data showed the *S*. *pneumoniae* serotype coverage of isolates from children who had undergone adenoidectomy for recurrent and/or persistent symptoms of URTI from south-east Poland by the currently available PCVs is high (54.4% and 66.4%) and similar to that reported in other European countries [10].

In the present study, 76.8% penicillin-nonsusceptible isolates were assigned to 7 clonal groups. Spain23F-1 (ST81) and Spain9V-3 (ST156) have been present in Poland since the second half of the 1990s [11, 33]. Since the introduction of a PCV7, a significant decrease in Spain23F-1 in some regions [34, 35] also in Poland the decrease from 19.1% to 6.8% of invasive and non-invasive PNSP isolates has been observed [11, 30]. In our study, ST81 clone constituted 16.1% of PNSP isolates. It might have arisen from specificity of the studied population: unvaccinated preschool children came from the region with a low vaccination rate and tested pneumococcal isolates were recovered from the upper respiratory tract (nasopharynx or adenoid samples). It is suggested that Spain23F-1 is a clone with low propensity for causing invasive diseases, and its intercontinental distribution has been facilitated by adaptation to colonization of the human nasopharynx and survival within [36]. High effectiveness at nasopharyngeal colonization gives a possibility of different exchange events with other pneumococci or viridans group streptococci, which could be a possible explanation for a large variety of transposons carried by representatives of ST81 observed in our study and studies performed by others [14, 26]. The Spain9V-3 complex disseminated in Poland between 1998–2002 and 2003–2005 from 22% to 47.5% of PNSP isolates [11, 33]. In our study, CG3 (ST156/SLV156) isolates represented only 5.4% of PNSP isolates, however, CG9 consisting of 3 PNSP isolates (ST143/SLV143) has turned out to be DLV of ST156. The dissemination of representatives of these two related clones Spain9V-3 and ST143 appears to be responsible for the increase in resistance observed in 2002 in Poland [33]. Surprisingly, 3 SLV/DLV of ST156 isolates were antibiotic sensitive and belonged to 15A serotype.

In addition to the presence of several international clones, we observed the most frequent CG12 comprising ST135/SLV135 MDR isolates of serotype 6B displaying the same genetic profile. ST135 has been isolated in several European countries, Turkey and Venezuela [14, 27, 33, 37] from healthy carriers and cases of invasive or non-invasive pneumococcal diseases. It is likely that ST135 could constitute a pandemic clone, therefore it should be investigated in detail. Clone 19F-ST87 observed in our was previously described as a frequently isolated clone in Spain [34], Portugal, Italy and Denmark but not in Poland (www.mlst.net).

In the present study, in the majority of chloramphenicol-resistant isolates (79.4%) ICESp23FST81-family elements were detected. Interestingly, all of the strains exclusively from CG2 (ST87/SLV87) with 19F serotype possessed marker genes positive for Tn5252. ICESp23FST81 elements were identified mostly in clonal groups (CG1, CG7, CG11). It can be the proof of clonal spreading strains carrying these elements in children population. The presence of Tn*5253*-family and ICESp23FST81-family elements has been investigated in clinical isolates of *S*. *pneumoniae* and proven to be frequent, especially among multidrug resistant strains, including internationally recognised pandemic clones [8, 38].

The main limitations of this study are the relatively small number of isolates and the fact that pneumococcal isolates were collected at a single center. However, our results may represent the national situation concerning non-invasive *S*. *pneumoniae* strains.

## Conclusions

Among non-invasive but disease-causing pneumococcal isolates from south-east Poland the low incidence of PMEN clones and high heterogeneity of clonal groups were observed. Our study describes a few MDR clones belonging to 6B and 19F serotypes potentially spreading among preschool children in the community. This observation emphasizes the role of clonal dissemination in the increase of resistance. A scarce decrease in the prevalence of vaccine serotypes is repercussion of a low vaccination rate in Poland. The introduction of a wide childhood vaccination program should hopefully lead to a reduction in the frequency of resistance. However, the appearance of non-vaccine MDR strains may compromise the effect of vaccination, therefore continuous surveillance of circulating isolates is still needed.

## Supporting Information

S1 Fig Diagram MLST—comparison of tested strains and 43 PMEN strains. (PDF)

S1 File Analysis of the similarity among tested isolates made by eBURSTv3 software. (PDF)

S2 File Analysis of the similarity among tested isolates and PMEN strains made by eBURSTv3 software. (PDF)

## References

[1] Bogaert D, de Groot R, Hermans PWM. *Streptococcus pneumoniae* colonization: the key to pneumococcal disease. The Lancet Infect Dis. 2004; 4:144–154. 14998500 10.1016/S1473-3099(04)00938-7

[2] De Lencastre H, Tomasz A. From ecological reservoir to disease: the nasopharynx, day care centres and drug-resistant clones of *Streptococcus pneumoniae*. J Antimicrob Chemother. 2002; 50 (Suppl S2):75–81. 12556436 10.1093/jac/dkf511

[3] Korona-Glowniak I, Niedzielski A, Kosikowska U, Grzegorczyk A, Malm A. Nasopharyngeal vs. adenoid cultures in children undergoing adenoidectomy: prevalence of bacterial pathogens, their interaction and risk factors. Epidemiol Infect. 2015; 143:821–830. 10.1017/S0950268814001460 25703401 PMC9507096

[4] Appelbaum PC. Resistance among *Streptococcus pneumoniae*: implications for drug selection. Clin Infect Dis. 2002; 34:1613–20. 12032897 10.1086/340400

[5] Rice LB. Tn916 family conjugative transposons and dissemination of antimicrobial resistance determinants. Antimicrob Agents Chemother. 1998; 42:1871–1877. 9687377 10.1128/aac.42.8.1871 PMC105703

[6] Del Grosso M, Camilli R, Iannelli F, Pozzi G, Pantosti A. The *mef*(E)-carrying genetic element (mega) of *Streptococcus pneumoniae*: insertion sites and association with other genetic elements. Antimicrob Agents Chemother. 2006; 50:3361–6. 17005818 10.1128/AAC.00277-06 PMC1610078

[7] Del Grosso M, Northwood JG, Farrell DJ, Pantosti A. The macrolide resistance genes *erm*(B) and *mef*(E) are carried by Tn*2010* in dual-gene *Streptococcus pneumoniae* isolates belonging to clonal complex CC271. Antimicrob Agents Chemother. 2007; 51:4184–4186. 17709465 10.1128/AAC.00598-07 PMC2151421

[8] Mingoia M, Tili E, Manso E, Varaldo PE, Montanari MP. Heterogenicity of Tn5253-like composite elements in clinical *Streptococcus pneumoniae* isolates. Antimcrob Agents Chemother. 2011; 55:1453–1459. 10.1128/AAC.01087-10 PMC3067147 21263055

[9] O’Brien KL, Wolfson LJ, Watt JP, Henkle E, Deloria-Knoll M, McCall N, et al The global burden of disease due to *Streptococcus pneumoniae* in children less than 5 years of age. Lancet 2009 374: 893–902. 19748398 10.1016/S0140-6736(09)61204-6

[10] Isaacman DJ, McIntosh ED, Reinert RR. Burden of invasive pneumococcal disease and serotype distribution among *Streptococcus pneumoniae* isolates in young children in Europe: impact of the 7-valent pneumococcal conjugate vaccine and considerations for the future conjugate vaccines. Int J Infect Dis. 2010; 14:e197–209. 10.1016/j.ijid.2009.05.010 19700359

[11] Sadowy E, Kuch A, Gniadkowski M, Hryniewicz W. Expansion and evolution of the *Streptococcus pneumoniae* Spain9V-ST156 clonal complex in Poland. Antimicrob Agents Chemother. 2010; 54:1720–1727. 10.1128/AAC.01340-09 20194703 PMC2863602

[12] Sirra L, Javala J, Tissari P, Vaara M, Kaijalainen T, Virolainen A. Clonality behind the increase of multidrug-resistance among non-invasive pneumococci in Southern Finland. Eur J Clin Microbiol Infect Dis. 2012; 31:867–871. 10.1007/s10096-011-1386-8 21870053

[13] Geng Q, Zhang T, Ding Y, Tao Y, Lin Y, Wang Y, et al Molecular characterization and antimicrobial susceptibility of *Streptococcus pneumoniae* isolated from children hospitalized with respiratory infections in Suzhou, China. PlosOne 2014; 9:e93752 10.1371/journal.pone.0093752 PMC3977860 24710108

[14] Calatayud L, Ardanuy C, Tubau F, Rolo D, Grau I, Pallarés R, et al Serotype and genotype replacement among macrolide-resistant invasive pneumococci in adults: mechanisms of resistance and association with different transposons. J Clin Microbiol. 2010; 48:1310–1316. 10.1128/JCM.01868-09 20147647 PMC2849543

[15] Llull D, López R, García E. Characteristic signatures of the *lytA* gene provide a basis for rapid and reliable diagnosis of *Streptococcus pneumoniae* infections. J Clin Microbiol. 2006; 44:1250–6. 16597847 10.1128/JCM.44.4.1250-1256.2006 PMC1448622

[16] Sutcliffe Y, Grebe T, Tait-Kamradt A, Wondrack L. Detection of erythromycin-resistant determinants by PCR. Antimicrob Agents Chemother. 1996; 40:2562–2566. 8913465 10.1128/aac.40.11.2562 PMC163576

[17] Montanari MP, Mingoia M, Giovanetti E, Veraldo PE. Phenotypes and genotypes of erythromycin-resistant Pneumococci in Italy. Clin Microb. 2003;4:428–431. 10.1128/JCM.41.1.428-431.2003 PMC149635 12517885

[18] Widdowson CA, Adrian PV, Klugman KP. Acquisition of chloramphenicol resistance by linearization and integration of the entire staphylococcal plasmid pC194 into chromosome of *Streptococcus pneumoniae*. Antimicrob Agents Chemother. 2000; 44:393–395. 10639367 10.1128/aac.44.2.393-395.2000 PMC89688

[19] Cochetti I, Tili E, Mingoia M, Varaldo PE, Montanari MP. *Erm*(B)-carring elements in tetracycline-resistant pneumococci and correspondence between Tn*1545* and Tn*6003*. Antimicrob Agents Chemother. 2008; 52:1285–1290. 10.1128/AAC.01457-07 18285489 PMC2292545

[20] Cochetti I, Tili E, Vecchi M, Manzin A, Mingoia M, Varaldo PE, et al New Tn*91*6-related elements causing *erm*(B)-mediated erythromycin resistance in tetracycline-susceptible pneumococci. J Antimicrob Chemother. 2007; 60:127–131. 17483548 10.1093/jac/dkm120

[21] Li Y, Tomita H, Lv Y, Liu J, Xue F, Zheng B, et al Molecular Characterization of erm(B)- and mef(E)-mediated erythromycin-resistant *Streptococcus pneumoniae* in China and complete DNA sequence of Tn2010. J Appl Microbiol. 2011; 110:254–265. 10.1111/j.1365-2672.2010.04875.x 20961364

[22] Izdebski R, Sadowy E, Fiett J, Grzesiowski P, Gniadkowski M, Hryniewicz W. Clonal diversity and resistance mechanism in tetracycline-nonsusceptible *Streptococcus pneumoniae* isolates in Poland. Antimicrob Agents Chemother. 2007; 51:1155–1163. 17210772 10.1128/AAC.01384-06 PMC1855514

[23] Enright MC, Spratt BG. A multilocus sequence typing scheme for *Streptococcus pneumoniae*: identification of clones associated with serious invasive disease. Microbiology 1998; 14:3049–3060. 10.1099/00221287-144-11-3049 9846740

[24] Korona-Glowniak I, Niedzielski A, Malm A, Niedzielska G. Serotypes and antibiotic resistance of *Streptococcus pneumoniae* from adenoids in preschool children with recurrent upper respiratory tract infections. Pol J Microbiol. 2013; 62:385–390. 24730133

[25] Vestrheim FD, Hoiby A, Aaberge IS, Caugant DA. Phenotypic and genotypic characterization of *Streptococcus pneumoniae* strains colonizing children attending day care centres in Norway. J Clin Microbiol. 2008; 46:2508–2518. 10.1128/JCM.02296-07 18524970 PMC2519506

[26] Xu X, Cai L, Xiao M, Kong F, Oftateh, Zhou F, et al Distribution of serotypes, genotypes, and resistance determinants among macrolide-resistant *Streptococcus pneumoniae* isolates. Antimicrob Agents Chemother. 2010; 54:1152–1159. 10.1128/AAC.01268-09 20065057 PMC2825966

[27] Quintero B, Araque, van der Geest-de Jongh C, Hermans PWM. Genetic diversity of 916-related transposons among drug-resistant *Streptococcus pneumoniae* isolates colonizing healthy children in Venezuela. Antimicrob Agents Chemother. 2011; 55:4930–4932. 10.1128/AAC.00242-11 21788464 PMC3187009

[28] Ardanuy C, Rolo D, Fenoll A, Tarrago D, Calatayud L, Liñares J. Emergence of a multidrug-resistant clone (ST320) among invasive serotype 19A pneumococci in Spain. J Antimicrob Chemother. 2009;64:507–10. 10.1093/jac/dkp210 19535383

[29] Moore MR, Gertz RE Jr, Woodbury RL, Barkocy-Gallagher GA, Schaffner W, Lexau C, et al Population snapshot of emergent Streptococcus pneumoniae serotype 19A in the United States, 2005. J Infect Dis. 2008;197:1016–27. 10.1086/528996 18419539

[30] Pillai DR, Shahinas D, Buzina A, Pollock RA, Lau R, Khairnar Ket, et al Genome-wide dissection of globally emergent multi-drug resistant serotype 19A Streptococcus pneumoniae. BMC Genomics. 2009;10:642 10.1186/1471-2164-10-642 20042094 PMC2807444

[31] Choi EH, Kim SH, Eun BW, Kim SJ, Kim NH, Lee J, et al Streptococcus pneumoniae serotype 19A in children, South Korea. Emerg Infect Dis. 2008;14:275–81. 10.3201/eid1402.070807 18258121 PMC2600206

[32] http://wwwold.pzh.gov.pl/oldpage/epimeld/index_p.html.

[33] Sadowy E, Izdebski R, Skoczynska A, Grzesiowski P, Gniadkowski M, Hryniewicz W. Phenotypic and molecular analysis of penicillin-nonsusceptible Streptococcus pneumoniae isolates in Poland. Antimicrob Agents Chemother. 2007; 51:40–47. 17043125 10.1128/AAC.01072-06 PMC1797676

[34] Calatayud L, Ardanuy C, Cercenado E, Fenoll AD, Bouza E, Pallarés R, et al Serotypes, clones, and mechanisms of resistance of erythromycin-resistant Streptococcus pneumoniae collected in Spain. Antimicrob Agents Chemother. 2007; 51:3240–3246. 17606677 10.1128/AAC.00157-07 PMC2043242

[35] Jacobs MR, Good CE, Beall B, Bajaksouzian S, Windau AR, Whitney CG. Changes in serotypes and antimicrobial susceptibility of invasive *Streptococcus pneumoniae* strains in Cleveland: a quarter century of experience. J Clin Microbiol. 2008; 46:982–990. 10.1128/JCM.02321-07 18234877 PMC2268364

[36] Croucher NJ, Walker D, Romero P, Lennard N, Paterson GK, Bason NC, et al Role of conjugative elements in the evolution of the multidrug-resistant pandemic clone *Streptococcus pneumoniae* Spain23F ST81. J Bacterial. 2009; 191:1480–1489. 10.1128/JB.01343-08 PMC2648205 19114491

[37] Zhou L, Ma X, Gao W, Yao KH, Shen AD, Yu SJ, et al Molecular characteristics of erythromycin-resistant *Streptococcus pneumoniae* from pediatric patients younger than five years in Beijing, 2010. BMC Microbiol. 2012; 12:228 10.1186/1471-2180-12-228 23043378 PMC3534231

[38] Henderson-Begg SK, Roberts AP, Hall LM. Diversity of putative Tn5253-like elements in *Streptococcus pneumoniae*. Int J Antimicrobial Agents. 2009; 33:364–367. 10.1016/j.ijantimicag.2008.10.002 19097761

