# Supplementary figures and images for: Molecular Epidemiology of Streptococcus pneumoniae Isolates from Children with Recurrent Upper Respiratory Tract Infections
Source: PLoS One. 2016 Jul 14;11(7):e0158909. doi: 10.1371/journal.pone.0158909 (PMC4945090; doi:10.1371/journal.pone.0158909)

**S1 Fig. Diagram MLST - comparison of tested strains and 43 PMEN strains.**

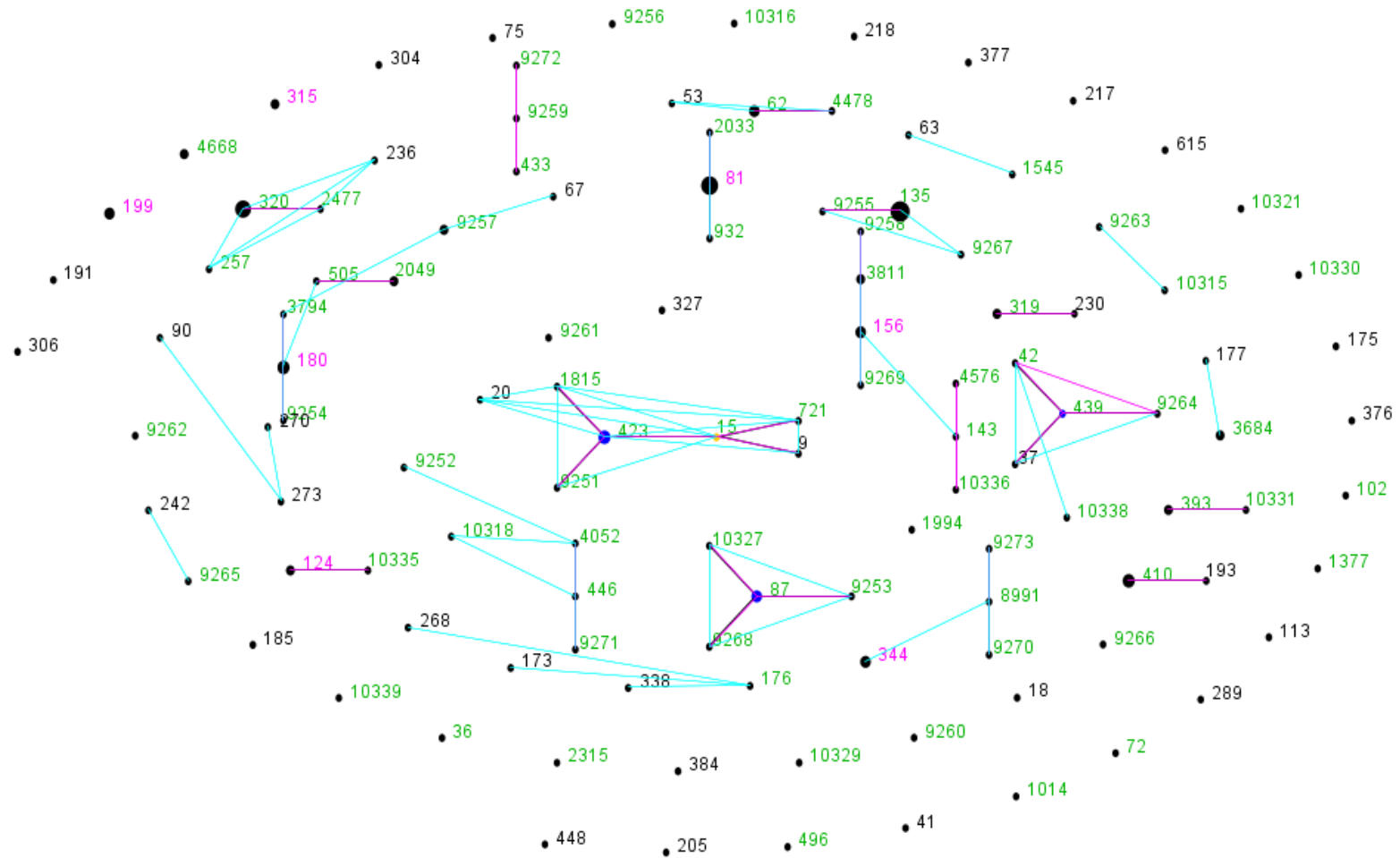

Supplement: S1 Fig — (PDF) [file pone.0158909.s001.pdf]
