# Supplementary material for: Molecular Epidemiology of Streptococcus pneumoniae Isolates from Children with Recurrent Upper Respiratory Tract Infections
Source: PLoS One. 2016 Jul 14;11(7):e0158909. doi: 10.1371/journal.pone.0158909 (PMC4945090; doi:10.1371/journal.pone.0158909)
Supplement: S1 File — (PDF) [file pone.0158909.s002.pdf]

S1 File. Analysis of the similarity among tested isolates made by eBURSTv3 software.

eBURST Report - Wed Aug 12 17:30:20 CEST 2015
No. isolates = 125 | No. STs = 82 | No. re-samplings for bootstrapping = 1000
No. loci per isolate = 7 | No. identical loci for group def = 6 | No. groups = 16

Group 1: No. Isolates = 8 | No. STs = 5 | Predicted Founder = 423

						Average		ST Bootstrap	
ST	FREQ	SLV	DLV	TLV	SAT	Distance		Group	Subgrp
423		4	3	1	0	0	1.25	63%	16%
15		1	2	2	0	0	1.5	19%	0%
1815		1	1	3	0	0	1.75	0%	0%
9251		1	1	2	1	0	2.0	0%	0%
721		1	1	2	1	0	2.0	0%	0%

Group 2: No. Isolates = 6 | No. STs = 4 | Predicted Founder = 87

						Average		ST Bootstrap	
ST	FREQ	SLV	DLV	TLV	SAT	Distance		Group	Subgrp
87		3	3	0	0	0	1.0	72%	11%
10327		1	1	2	0	0	1.66	0%	0%
9253		1	1	2	0	0	1.66	0%	0%
9268		1	1	2	0	0	1.66	0%	0%

Group 3: No. Isolates = 6 | No. STs = 4 | Predicted Founder = Multiple Candidates

						Average		ST Bootstrap	
ST	FREQ	SLV	DLV	TLV	SAT	Distance		Group	Subgrp
3811		2	2	1	0	0	1.33	27%	0%
156		2	2	1	0	0	1.33	29%	0%
9258		1	1	2	0	0	1.66	0%	0%
9269		1	1	2	0	0	1.66	0%	0%

Group 4: No. Isolates = 3 | No. STs = 3 | Predicted Founder = Multiple Candidates

						Average		ST Bootstrap	
ST	FREQ	SLV	DLV	TLV	SAT	Distance		Group	Subgrp
9259		1	2	0	0	0	1.0	9%	0%
9272		1	2	0	0	0	1.0	7%	0%
433		1	2	0	0	0	1.0	13%	0%

Group 5: No. Isolates = 5 | No. STs = 3 | Predicted Founder = 180

						Average		ST Bootstrap	
ST	FREQ	SLV	DLV	TLV	SAT	Distance		Group	Subgrp
180		3	2	0	0	0	1.0	29%	0%
9254		1	1	1	0	0	1.5	0%	0%
3794		1	1	1	0	0	1.5	0%	0%

Group 6: No. Isolates = 3 | No. STs = 3 | Predicted Founder = 8991

						Average		ST Bootstrap	
ST	FREQ	SLV	DLV	TLV	SAT	Distance		Group	Subgrp
8991		1	2	0	0	0	1.0	32%	0%
9273		1	1	1	0	0	1.5	0%	0%
9270		1	1	1	0	0	1.5	0%	0%

Group 7: No. Isolates = 9 | No. STs = 3 | Predicted Founder = 81

ST	FREQ	SLV	DLV	TLV	SAT	Average Distance		ST Bootstrap Group	Subgrp
81		7	2	0	0	0	1.0	34%	0%
2033		1	1	1	0	0	1.5	0%	0%
932		1	1	1	0	0	1.5	0%	0%

Group 8: No. Isolates = 3 | No. STs = 3 | Predicted Founder = Multiple Candidates

ST	FREQ	SLV	DLV	TLV	SAT	Average Distance		ST Bootstrap Group	Subgrp
42		1	2	0	0	0	1.0	7%	0%
439		1	2	0	0	0	1.0	8%	0%
9264		1	2	0	0	0	1.0	15%	0%

Group 9: No. Isolates = 3 | No. STs = 3 | Predicted Founder = Multiple Candidates

ST	FREQ	SLV	DLV	TLV	SAT	Average Distance		ST Bootstrap Group	Subgrp
4576		1	2	0	0	0	1.0	7%	0%
143		1	2	0	0	0	1.0	7%	0%
10336		1	2	0	0	0	1.0	15%	0%

Group 10: No. Isolates = 3 | No. STs = 3 | Predicted Founder = 446

ST	FREQ	SLV	DLV	TLV	SAT	Average Distance		ST Bootstrap Group	Subgrp
446		1	2	0	0	0	1.0	31%	0%
9271		1	1	1	0	0	1.5	0%	0%
4052		1	1	1	0	0	1.5	0%	0%

Group 11: No. Isolates = 8 | No. STs = 2 | Predicted Founder = None

ST	FREQ	SLV	DLV	TLV	SAT	Distance	
320		7	1	0	0	0	1.0
2477		1	1	0	0	0	1.0

Group 12: No. Isolates = 11 | No. STs = 2 | Predicted Founder = None

ST	FREQ	SLV	DLV	TLV	SAT	Distance	
135		10	1	0	0	0	1.0
9255		1	1	0	0	0	1.0

Group 13: No. Isolates = 3 | No. STs = 2 | Predicted Founder = None

ST	FREQ	SLV	DLV	TLV	SAT	Distance	
2049		2	1	0	0	0	1.0
505		1	1	0	0	0	1.0

Group 14: No. Isolates = 2 | No. STs = 2 | Predicted Founder = None

ST	FREQ	SLV	DLV	TLV	SAT	Distance

124	1	1	0	0	0	1.0
10335	1	1	0	0	0	1.0

Group 15: No. Isolates = 4 | No. STs = 2 | Predicted Founder = None

ST	FREQ	SLV	DLV	TLV	SAT	Distance
62	3	1	0	0	0	1.0
4478	1	1	0	0	0	1.0

Group 16: No. Isolates = 3 | No. STs = 2 | Predicted Founder = None

ST	FREQ	SLV	DLV	TLV	SAT	Distance
393	2	1	0	0	0	1.0
10331	1	1	0	0	0	1.0

Singletons: size 36
10329
9257
9256
10321
9252
319
410
315
10318
10316
10315
176
3684
102
36
1377
2315
257
1014
4668
344
72
496
10339
10338
9267
9266
9265
9263
10330
9262
9261
1545
9260
199
1994
