# Supplementary material for: Molecular Epidemiology of Streptococcus pneumoniae Isolates from Children with Recurrent Upper Respiratory Tract Infections
Source: PLoS One. 2016 Jul 14;11(7):e0158909. doi: 10.1371/journal.pone.0158909 (PMC4945090; doi:10.1371/journal.pone.0158909)
Supplement: S2 File — (PDF) [file pone.0158909.s003.pdf]

S2 File. Analysis of the similarity among tested pneumococcal isolates and PMEN strains (highlighted) made by eBURSTv3 software.

eBURST Report - Thu Oct 15 16:56:52 CEST 2015
No. isolates = 168 | No. STs = 118 | No. re-samplings for bootstrapping = 1000
No. loci per isolate = 7 | No. identical loci for group def = 6 | No. groups = 18

Group 1: No. Isolates = 9 | No. STs = 6 | Predicted Founder = 423

ST	FREQ	SLV	DLV	TLV	SAT	Average Distance		ST Bootstrap Group	Subgrp
423		4	3	2	0	0	1.4	49%	21%
15		1	3	2	0	0	1.4	50%	19%
1815		1	1	3	1	0	2.0	0%	0%
721		1	1	3	1	0	2.0	0%	0%
9251		1	1	2	2	0	2.2	0%	0%
9		1	1	2	2	0	2.2	0%	0%

Group 2: No. Isolates = 6 | No. STs = 4 | Predicted Founder = 87

ST	FREQ	SLV	DLV	TLV	SAT	Average Distance		ST Bootstrap Group	Subgrp
87		3	3	0	0	0	1.0	74%	15%
10327		1	1	2	0	0	1.66	0%	0%
9253		1	1	2	0	0	1.66	0%	0%
9268		1	1	2	0	0	1.66	0%	0%

Group 3: No. Isolates = 7 | No. STs = 4 | Predicted Founder = 156

ST	FREQ	SLV	DLV	TLV	SAT	Average Distance		ST Bootstrap Group	Subgrp
156		3	2	1	0	0	1.33	26%	0%
3811		2	2	1	0	0	1.33	29%	0%
9258		1	1	2	0	0	1.66	0%	0%
9269		1	1	2	0	0	1.66	0%	0%

Group 4: No. Isolates = 4 | No. STs = 4 | Predicted Founder = 439

ST	FREQ	SLV	DLV	TLV	SAT	Average Distance		ST Bootstrap Group	Subgrp
439		1	3	0	0	0	1.0	61%	14%
42		1	2	1	0	0	1.33	4%	0%
9264		1	2	1	0	0	1.33	9%	0%
37		1	1	2	0	0	1.66	0%	0%

Group 5: No. Isolates = 3 | No. STs = 3 | Predicted Founder = Multiple Candidates

ST	FREQ	SLV	DLV	TLV	SAT	Average Distance		ST Bootstrap Group	Subgrp
9259		1	2	0	0	0	1.0	7%	0%
9272		1	2	0	0	0	1.0	10%	0%
433		1	2	0	0	0	1.0	15%	0%

Group 6: No. Isolates = 6 | No. STs = 3 | Predicted Founder = 180

Average ST Bootstrap

ST	FREQ	SLV	DLV	TLV	SAT	Distance		Group	Subgrp
180		4	2	0	0	0	1.0	32%	0%
9254		1	1	1	0	0	1.5	0%	0%
3794		1	1	1	0	0	1.5	0%	0%

Group 7: No. Isolates = 3 | No. STs = 3 | Predicted Founder = 8991

						Average		ST Bootstrap	
ST	FREQ	SLV	DLV	TLV	SAT	Distance		Group	Subgrp
8991		1	2	0	0	0	1.0	31%	0%
9273		1	1	1	0	0	1.5	0%	0%
9270		1	1	1	0	0	1.5	0%	0%

Group 8: No. Isolates = 10 | No. STs = 3 | Predicted Founder = 81

						Average		ST Bootstrap	
ST	FREQ	SLV	DLV	TLV	SAT	Distance		Group	Subgrp
81		8	2	0	0	0	1.0	32%	0%
2033		1	1	1	0	0	1.5	0%	0%
932		1	1	1	0	0	1.5	0%	0%

Group 9: No. Isolates = 3 | No. STs = 3 | Predicted Founder = Multiple Candidates

						Average		ST Bootstrap	
ST	FREQ	SLV	DLV	TLV	SAT	Distance		Group	Subgrp
4576		1	2	0	0	0	1.0	7%	0%
143		1	2	0	0	0	1.0	8%	0%
10336		1	2	0	0	0	1.0	17%	0%

Group 10: No. Isolates = 3 | No. STs = 3 | Predicted Founder = 446

						Average		ST Bootstrap	
ST	FREQ	SLV	DLV	TLV	SAT	Distance		Group	Subgrp
446		1	2	0	0	0	1.0	31%	0%
9271		1	1	1	0	0	1.5	0%	0%
4052		1	1	1	0	0	1.5	0%	0%

Group 11: No. Isolates = 8 | No. STs = 2 | Predicted Founder = None

ST	FREQ	SLV	DLV	TLV	SAT	Distance	
320		7	1	0	0	0	1.0
2477		1	1	0	0	0	1.0

Group 12: No. Isolates = 11 | No. STs = 2 | Predicted Founder = None

ST	FREQ	SLV	DLV	TLV	SAT	Distance	
135		10	1	0	0	0	1.0
9255		1	1	0	0	0	1.0

Group 13: No. Isolates = 3 | No. STs = 2 | Predicted Founder = None

ST	FREQ	SLV	DLV	TLV	SAT	Distance	
2049		2	1	0	0	0	1.0
505		1	1	0	0	0	1.0

Group 14: No. Isolates = 3 | No. STs = 2 | Predicted Founder = None

ST	FREQ	SLV	DLV	TLV	SAT	Distance	
124		2	1	0	0	0	1.0
10335		1	1	0	0	0	1.0

Group 15: No. Isolates = 3 | No. STs = 2 | Predicted Founder = None

ST	FREQ	SLV	DLV	TLV	SAT	Distance	
319		2	1	0	0	0	1.0
230		1	1	0	0	0	1.0

Group 16: No. Isolates = 5 | No. STs = 2 | Predicted Founder = None

ST	FREQ	SLV	DLV	TLV	SAT	Distance	
410		4	1	0	0	0	1.0
193		1	1	0	0	0	1.0

Group 17: No. Isolates = 4 | No. STs = 2 | Predicted Founder = None

ST	FREQ	SLV	DLV	TLV	SAT	Distance	
62		3	1	0	0	0	1.0
4478		1	1	0	0	0	1.0

Group 18: No. Isolates = 3 | No. STs = 2 | Predicted Founder = None

ST	FREQ	SLV	DLV	TLV	SAT	Distance	
393		2	1	0	0	0	1.0
10331		1	1	0	0	0	1.0

Singletons: size 66

10329
9257
384
9256
289
10321
9252
615
185
53
315
10318
377
218
10316
376
217
10315
273
113
177

270
176
175
173
3684
41
306
304
205
268
102
36
1377
2315
90
257
1014
4668
448
20
344
242
18
75
72
338
496
10339
10338
236
9267
9266
9265
9263
10330
9262
9261
1545
9260
199
67
191
63
1994
327
